# Sentinel-lymph-node mapping with indocyanine green in robotic-assisted laparoscopic surgery for early endometrial cancer: a retrospective analysis

**Published:** 2020-03-27

**Authors:** V Cela, C Sergiampietri, ME Rosa Obino, G Bifulco, P Giovanni Artini, F Papini

**Affiliations:** Department of Experimental and Clinical Medicine, Division of Obstetrics and Gynecology, University of Pisa, Pisa, Italy;; Department of Neuroscience, Reproductive Sciences and Dentistry, University of Naples Federico II, Naples, Italy.

**Keywords:** Gynecological malignancy, lymphadenectomy, robotic surgery, surgical staging, indocyanine green

## Abstract

**Background:**

The therapeutic value of lymphadenectomy in early stage endometrial cancer (EC) is still debated. Sentinel-lymph-node identified with indocyanine green (ICG) can replace lymphadenectomy in the staging of endometrial cancer minimizing the potential morbidity of a complete lymphadenectomy. The aim of this study was to analyze our initial experience using indocyanine green for sentinel-lymph-node mapping in a minimally robotic-assisted laparoscopic approach with Da Vinci XI near-infrared (NIR) fluorescence imaging system.

**Methods:**

A total of 23 patients who underwent robot-assisted laparoscopic surgery with the Da Vinci Xi Surgical System (Intuitive Surgical, Sunnyvale, CA, USA) with NIR imaging and ICG fluorescence detection for early stage EC were retrospectively analyzed.

**Results:**

Sentinel-lymph-node mapping was achieved in 18 patients for a detection rate of 78.26%, bilateral pelvic detection was possible in 14 patients (60.9%) and no sentinel-lymph-node mapping was noted in 4 patients (17.4%). We compared 11 patients (Group 1) at intermediate and high- risk of recurrence who underwent sentinel-lymph- node mapping and pelvic lymphadenectomy and 12 patients (Group 2) at low risk of recurrence who underwent only sentinel-lymph-node mapping. A statistically significant difference was found for the average operation time and for the hospital stays.

**Conclusions:**

The high detection rate, absence of intraoperative or postoperative complications, the short time required for mapping and removal of the sentinel-lymph-nodes and the short duration of the hospital stay, support performing sentinel-lymph-node in all women with early endometrial cancer.

## Introduction

Endometrial cancer (EC) is the most frequent gynecological malignancy. About 80% of cases are diagnosed at an early stage. Depending on its histological characteristics, a 5-year overall survival can vary from 42% to 92% in International Federation of Gynecology and Obstetrics (FIGO) stage I disease. (Ballester et al., 2017). Regional lymph node status is important for correct staging, as metastasis to regional lymph nodes changes FIGO status from stage 1 to stage 3 C (Hagen et al., 2016; Gilani et al., 2014; Corrado et al., 2016). Even though one prospective randomized trial of pelvic lymphadenectomy in EC failed to demonstrate survival benefits (Panici et al., 2008), the therapeutic value of lymphadenectomy is still debated (Chan et al., 2006). The risk of lymph node metastasis in early-stage, low-grade tumors is relatively low, and the potential morbidity of lymphadenectomy may outweigh population-based clinical benefits. It has been shown that well-differentiated tumors have a risk of metastasis in pelvic and paraaortic lymph nodes of 3% and 2% respectively and a tumor confined to the endometrium has a global risk of lymph node-metastasis of 1% (Holloway et al., 2017). The prognostic importance of regional lymphadenectomy is widely discussed (Panici et al., 2008; Kitchener et al., 2009; Naumann et al., 2012) and there is still no consensus on the role of lymphadenectomy in the treatment of EC (Fotopoulou et al., 2015). Sentinel lymphonodectomy has gained more attention in EC as surgeons try to minimize treatment related morbidity while providing adequate surgical staging. The aim of this study was to analyze retrospectively our initial experience using indocyanine green(ICG) for sentinel lymph node (SLN) mapping in a minimally invasive, robotic-assisted laparoscopic approach with the DaVinci XI near-infrared (NIR)/ fluorescence imaging system in women with apparent early stage EC.

## Materials and methods

### 

Twenty-three patients with apparent early stage EC were enrolled in a retrospective observational study about SLN detection. Surgery was performed between December 2015 and December 2017. All patients gave their written informed consent. The study was approved by the institutional review board. Full ethical review was not required owing to the retrospective nature of the study. Robot- assisted laparoscopic surgery was performed with the Da Vinci Xi Surgical System (Intuitive Surgical, Sunnyvale, CA, USA), with NIR imaging and ICG fluorescence detection.

All patients with histologically confirmed EC underwent preoperative imaging with computerized tomography (CT) of the thorax and abdomen/pelvis, as well as an expert transvaginal ultrasonography to assess myometrial and cervical invasion. They underwent simple robotic-assisted laparoscopic hysterectomy, bilateral salpingo- oophorectomy and SLN mapping, according to the traditional preoperative risk categories (based on histopathologic type and grade, and depth of myometrial tumor infiltration) and to current recommendations (Ballester et al., 2017). A full pelvic lymphnode staging following removal of the SLNs was performed in all intermediate and high-risk EC patients. In low-risk patients the procedure was restricted to SLN removal. For each patient a 25 mg vial of ICG powder (Acorn Pharmaceuticals, Lake Forest, IL, USA) was diluted into 20 ml of aqueous sterile water. Immediately before docking of the robot, the cervix was slowly injected with a total of 4 ml of ICG (1,25 mg/mL) at the 9 and 3 o’clock positions (1 ml superficially under the mucosa and 1 ml 1 cm deeply into the cervical stroma on each side with a 22-gauge spinal needle). All these procedures were performed by two collaborating surgeons. After opening of the retroperitoneal space, the NIR system was activated, identifying the lymphatic trunks leading to the marked SLNs. Then, the lymph node stations were dissected gently to minimize bleeding and rupture of lymphatic channels. The position of each SLN was recorded on a standardized form. The surgeon was able to switch the NIR on and off as needed.

All operations were performed by the same surgeon. In some cases SLN mapping in the pelvis was not achieved or only seen on one side. An expert gynecological oncology pathologist skilled in the analysis of SLNs evaluated all surgical specimen and SLNs. All lymph nodes were routinely sectioned and stained with hematoxylin and eosin (H&E). The protocol used for the SLN assessment followed the recommendations which have been previously published by Kim et al. (2013). SLNs were initially examined by routine H&E staining and subsequent ultrastaging was performed if the initial H&E assessment was negative. SLN ultrastaging was performed by cutting two adjacent 5-μm sections at each of two levels, 50-μm apart, from each paraffin block lacking metastatic carcinoma. At each level, one slide was stained with H&E and with immunohistochemistry (IHC) using the anti- cytokeratin AE1:AE3 for a total of five slides per block. SLNs carrying low-volume, ultrastage- detected disease included both micrometastases and isolated tumor cells (Jamison et al., 2014). Macrometastasis in lymph nodes was defined as tumor cell infiltrates >2 mm. Micrometastasis in lymph nodes was defined as a focus of metastatic tumor cells > 0,2 mm and < 2 mm, whereas isolated tumor cells were defined as microscopic clusters and single cells measuring < 0,2 mm.

### Statistical analysis

Descriptive and comparative statistics were calculated using the JMP Pro 11.2 (SAS Institute Inc., Cary, NC). The categorical variables are represented as absolute values and/or percentages of the total and analysed by Fisher’s exact test. The continuous variables are represented as mean values ± standard deviation and analysed by Student’s T test, Mann-Whitney test and Kruskal-Wallis test. Correlation analysis was performed using the Spearman test. Statistical significance was defined as p< 0.05.

## Results

We retrospectively analyzed 23 patients with apparent early stage EC. For all patients robotic procedures were completed successfully without conversion to laparotomy; at the end of the procedure they all had vaginal extraction of the hysterectomy specimen. The demographic characteristics are summarized in Table I. Clinical-pathological characteristics are summarized in Table II.

**Table I:** — Demographic characteristics.

Age (average ± SD)	61.3+8.5
BMI Kg/m2 (average ± SD)	27.9+4.3
Symptoms	100% bleeding
Pap-test	100% negative
Pre-surgery histological examination	100% Type 1
Markers (CA125/HE4)	100% negative
Pre-surgery radiological exam (TC/PET)	100% negative

SD: standard deviation

**Table II:** — Clinical-pathological characteristics.

Histology (Definitive HA)	
Type 1	23 (100%)
Type 2	0
Stage (Definitive HA)	
IA	16/23 (69.6%)
IB	4/23 (17.4%)
II	1/23 (4.3%)
IIIA	1/23 (4.3%)
IIIIC1	1/23 (4.3%)
Grade (Definitive HA)	
1	11/23 (47.83%)
2	9/23 (39.13%)
3	3/23 (13.04 %)

HA: histological analysis

Definitive histological analysis (HA) for all patients (23/23, 100%) showed Type 1 EC. Final stage assessment was: IA (16/23, 69.6%), IB (4/23, 17.4%), II (1/23, 4.3%), IIIA (1/23, 4.3%), IIIC1 (1/23, 4.3%). The grade (G) at the definitive HA was: G1 (11/23, 47.83%), G2 (9/23, 39.13%), G3 (3/23, 13.04%).

Surgery and post-surgery characteristics are summarized in Table III. The average operating time (OT) defined from placement of vaginal manipulator to skin closure was 160.5 ± 57.78 min. The average time from intracervical ICG injection to SLN identification was 37.83 ± 23.82 min in the left hemipelvis and 30.48 ± 24.35min in the right hemipelvis. Median estimated blood loss (EBL) was 74.34 ± 45.90 ml with no blood transfusions administered. The average hospital stay (HS) was 2.52 ± 0.66 days. No significant intra-operative, early or late post-operative complications occurred in any of the patients (23/23, 100%). Specifically, no complications occurred related to the ICG dye.

**Table III:** — Surgery and post-surgery characteristics.

OT (average ± SD)	160.5 ± 57.78 min
Time for SLN identification (average ± SD)	37.83 ± 23.82 in the left hemipelvis 30.48 ± 24.35 in the right hemipelvis

OT: operation time. SD: standard deviation. SLN: Sentinel-lymph-node. EBL: estimated blood loss.

In total, SLN mapping was successfully achieved in 18 patients for a detection rate (DR) of 78,26%; bilateral pelvic detection was seen in 14 patients (60.9%). No SLN mapping was observed in 4 patients (17.4%). The frequency of unilateral SLN detection (n=5/23, 21.7%) was different for the right (1/5, 20%) and the left (4/5, 80%) pelvic side. Among the 4 patients without SLN detection 3 were obese with BMI > 30. The patient with unsuccessful mapping with a BMI < 30 had had a previous conization.

Patients with successful bilateral mapping had a median BMI of 27.5 ± 3.67 kg/m2, patients with unilateral mapping had a median BMI of 29 ± 7.07 kg/m2 and patients with unsuccessful mapping had a median BMI of 28.25 ± 3.5 kg/m (p=.936). The total number of the SLNs detected was 53. The median number of SLN identified on the left side was 1.35 ± 1,07, the median number of SLN identified on the right side was 0.96 ± 1.02. SLNs were located primarily on the external iliac (37/53, 39.8%), followed by the internal iliac (6/53, 11.3%) common iliac (6/53, 11.3%) and obturator vessels (3/53, 5.7%) and the presacral region (1/53, 1.9%). Among the included patients, there were 6 patients with an intermediate-risk profile (6/23, 26.1 %), 5 patients with a high-risk profile (5/23, 21.7%) and 12 patients with low-risk profile (12/23, 52.2%). All the 23 patients preoperatively allocated to the low, intermediate and high- risk groups respectively, at the final histology they were confirm to the respective preoperative risk-group. We compared the group of 11 patients at intermediate and high- risk of recurrence who underwent SLN mapping and pelvic lymphadenectomy (Group 1) and the group of 12 patients at low risk of recurrence who underwent only SLN mapping (Group 2). In group 1, one micrometastasis was identified in 1 patient in the SLNs (1/11, 9%) and for the same patient the final histological examination detected a metastatic disease in the pelvic lymph nodes of the same hemipelvis. The other 10 patients of this group had negative SLNs and also negative nodes at the final histological examination of the complete pelvic lymph nodes. Therefore, for Group 1, we were able to calculate the Cohen’s Kappa coefficient demonstrating the perfect statistical agreement between the histological results of SLNs and the final histological results of pelvic lymph nodes (Cohen’s Kappa=1) and the reliability of the techniques. The median number of pelvic lymph node removed in Group 1 was 12.9 ± 8.5. The characteristics and the comparison between the two groups are described in Table IV. As expected, a significant difference was seen for the operation time (OT) (median OT Group1 193.6 ± 66.8 min and median OT Group 2 130.4 min ± 23.1; p = 0.011) and for the HS (median HS in Group 1 was 3 ± 0.6 days and median HS in Group 2 was 2.1 ± 0.3 min; p = .001). No statistically significant difference was seen for blood loss (median EBL Group1 86.4 ± 32.3 ml and median EBL Group2 63.3 ± 54.7 ml; p= 0.23). We also analyzed the correlation between the detection time and surgeon experience as well as the detection time and the BMI, but no significant correlations were found (Table V).

**Table IV:** — Characteristics and comparison between Group 1 and Group 2.

	Group 1	Group 2	p
Age (average ± SD)	62.6 ± 8.5	60.2 ± 8.8	p = .5
BMI (average + SD)	27.6 Kg/m 2 ± 4.5	28.3 Kg/m 2 ± 4.5	p = .75
Previous pregnancy (average + SD)	1.4 ± .9	1.3 ± .9	p = .76
Previous caesarean section (average + SD)	.5 ± .7	.4 ± .8	p = .9
ASA (average + SD)	2.5 ± .5	2.4 ± .5	p = .56
Concomitant medical diseases	5/11 (45.5%)	4/12 (33.3%)	p =.57
Previous tumor	1/11 (9%)	0/12 (0%)	p =.34
Previous abdominal laparotomy or laparoscopy	4/11 (36.4%)	3/12 (25%)	p =.66
Presence of contemporaneous tumor	1/11 (9%)	0/12 (0%)	p = .34
Execution of other surgical procedures	3/11 (27.3%)	3/12 (25%)	p = .9
OT (average + SD)	193.6 ± 66.8 min	130.4 ± 23.1 min	p = .011
HS (average + SD)	3 ± .6 days	2.1 ± .3 days	p = .001
EBL (average + SD)	86.4 ± 32.3 ml	63.3 ± 54.7 ml	p = .23

SD: standard deviation. BMI: body mass index. ASA: American Society of Anesthesiologists. OT: operation time. HS: hospital stays. EBL: estimated blood loss.

**Table V:** — Detection time correlation.

Variable	by Variable	Spearman ρ	Prob> ρ
Detection time SLN left (min)	Experience (days)	.296	.219
Detection time SLN right (min)	Experience (days)	.116	.668
BMI (Kg/m2)	Detection time SLN left (min)	.347	.146
BMI (Kg/m2)	Detection time SLN right (min)	-.245	.360

## Discussion

The recommendations of the European Society for Medical Oncology (ESMO), the European Society for Radiotherapy & Oncology (ESTRO) and the European Society of Gynaecological Oncology (ESGO) in 2016 and 2017 (Colombo et al., 2016; Ballester et al., 2017) reported that lymphadenectomy is not recommended in women with a low-risk of EC (Type 1 EC/stage FIGO IA/ grade 1-2). For patients with an intermediate-risk EC (Type 1 EC/stage FIGO IA/grade 3, Type 1 EC/stage FIGO IB/grade 1–2) lymphadenectomy remains an option but there is no proven benefit in terms of survival, especially for patients with FIGO IA grade 3 tumor. For patients at high risk of recurrence (Type 1 EC /stage FIGO IB/grade 3, Stage FIGO >= II, Type 2 EC), lymphadenectomy is recommended. SLN mapping has been described for EC since 2006 (Rossi et al., 2017). The National Comprehensive Cancer Network (NCCN) guidelines stated that SLN mapping may be considered as part of the surgical staging in apparent stage I disease, adhering to a strict algorithm defined by Barlin et al. (2012). Before the SLN era, the reported rate of lymph node metastasis in low-intermediate risk patients was 5%; by adding SLN mapping to current surgical staging procedures the likelihood of detecting metastatic cancer cells in regional lymph nodes could be increased. An additional benefit of incorporating pathologic ultrastaging of SLN is the detection of micrometastasis (Khoury-Collado et al., 2011). Ballester et al. (2013) demonstrated that SLN mapping identified metastasis in 12.5% of low risk patients and 21.2% of intermediate risk patients. Buda et al. (2016) showed that SLN mapping using ICG had a higher detection rate (DR) compared to other modalities. Several trials described the use of ICG dye for SLN mapping in EC in conjunction with the Da Vinci robotic fluorescence system (Intuitive Surgical, Sunnyvale, CA) demonstrating the reliability and safety of this method (Rossi et al., 2012; Rossi et al., 2013; Holloway et al., 2012; Sinno et al., 2014; Jewell et al., 2014). Furthermore cervical ICG injection achieves a higher SLN detection rate and a similar anatomic nodal distribution as hysteroscopic endometrial injection for SLN mapping in patients with endometrial cancer (Rossi et al., 2013). The use of SLN is an acceptable solution between “understaging” versus “overtreatment” of women with early stage EC. According to the most recent NCCN guidelines, a published SLN algorithm incorporating ultrastaging may be considered for the surgical staging of selected patients with apparent uterine-confined endometrial cancer (Koh et al., 2018).

Surely, the potential importance of low volume nodal metastasis detected by pathological ultrastaging of SLNs has added another important rationale for staging with this technique.

In this study we performed ICG intra-cervical injection with NIR imaging to detect the fluorescent dye during robot-assisted surgery (Buda et al., 2016; Lin et al., 2017).

Our favourable results agree with other series reported in the literature (Rossi et al., 2012; Rossi et al., 2013; Holloway et al., 2012; Sinno et al., 2014; Jewell et al., 2014), with an overall DR of 78.26% and a bilateral DR of 60.9%. We have achieved similar results to How et al. (2015) that reported 87% overall and 65% bilateral ICG DR, although our case series is smaller.

In our study we were not able to identify a correlation between obesity and detection rate. Concerning this matter statements in the literature are controversial. Some studies showed that obesity reduces ICG SLN mapping (Khoury-Collado et al., 2011; Lin et al., 2017) but a large meta-analysis showed that BMI ≥ 30 kg/m2 is not significantly associated with detection rates (Bodurtha et al., 2017; Corrado et al, 2018).

Regarding SLN mapping after previous conization, the majority of studies and meta-analysis regarding cervical cancer showed that conization has not been associated with a lower detection rate while a study (using radiotracer/blue dye) reported a significantly lower detection rate following conization (Buda et al., 2017).

In our limited experience, to perform the ICG cervical injection in a cervix after conization is technically more difficult and we often found widespread dye distribution in the pelvis.

In our experience the most frequent site of SLN mapping is the external iliac location. This is similar to the study of Geppert at al. (2017). In low risk patients, who do not require lymphadenectomy according to literature (Barlin et al., 2012), we performed only SLN mapping without pelvic lymphadenectomy. In Group 2 (intermediate and high risk) we performed SLN mapping with pelvic lymphadenectomy. In Group 2 we were able to demonstrate the validity and the reliability of the techniques of SLN mapping (Cohen’s Kappa = 1), because all the patients underwent pelvic lymphadenectomy.

We compared the two groups that were homogeneous for age, BMI, previous surgeries medical disease. As expected, comparing the groups, there is a significant statistically difference for the OT and the HS. We can therefore state that performing only the SLN mapping allows the patients to undergo a shorter surgery and to have a shorter HS with a consequent faster recovery. Performing SNL in our case series was not found to be associated with complications and was a manageable technique with an acceptable learning curve.

## Conclusion

In conclusion, relying on our results, we can confirm reproducibility of SLN mapping and effectiveness in finding metastases as well as adequate safety.
